# The Effects of Medicaid Coverage for School‐Based Health Services on the Supply of School‐Based Healthcare Clinics: Evidence From the “Free Care Rule” Reversal and FQHCs

**DOI:** 10.1111/1475-6773.14452

**Published:** 2025-02-12

**Authors:** Lindsey Rose Bullinger, Scarlette Jiajing Shi

**Affiliations:** ^1^ School of Public Policy at Georgia Institute of Technology Atlanta Georgia USA; ^2^ Georgia State University & Georgia Institute of Technology Atlanta Georgia USA

**Keywords:** children, Medicaid, poverty, school health services

## Abstract

**Objective:**

To determine whether Medicaid coverage of healthcare services provided within schools affects the supply of school‐based health clinics.

**Data Sources and Study Setting:**

We combine nationwide data spanning 2012–2020 on Federally Qualified Health Centers delivering health services within school settings with state policy information on Medicaid expansion to school‐based healthcare.

**Study Design:**

Until 2014, the federal “Free Care Rule” restricted school‐based health centers from using Medicaid funding to provide health services unless these services were part of the student's Individual Education Plan. In 2014, the Centers for Medicare & Medicaid Services reversed the free care rule, allowing states to opt in to authorizing Medicaid reimbursement for comprehensive health services delivered in schools to Medicaid‐enrolled students. We compared the number of federally qualified school‐based health centers in states that expanded their Medicaid policies in response to the free care rule reversal to that of states that did not expand their Medicaid programs to reimburse for school‐based health services in a staggered difference‐in‐differences analysis. We also adjusted the analysis for other important measures that may independently affect school‐based healthcare provision and funding decisions.

**Data Collection:**

The number of health centers delivering services in schools comes from the Health Resources & Services Administration. State Medicaid coverage policy information comes from the Healthy Schools Campaign.

**Principal Findings:**

Expanding Medicaid coverage to comprehensive school‐based healthcare increased the number of Federally Qualified Health Centers providing services in school settings by about 13.42 (*p* = 0.035, 95% CI: 0.94, 25.90). This increase represents a relative rise of 39%. However, these results are sensitive to specifications.

**Conclusions:**

Local educational entities, school health providers, and public health professionals are responsive to financial incentives to provide services to Medicaid‐enrolled children within schools. Recent federal legislation has the potential to increase financial incentives and offer complementarities to prior state‐level policy changes.

**Summary**

- What is known on this topic
  ○ In 2014, a longstanding federal policy changed, allowing states to expand their Medicaid programs to cover school‐based services for Medicaid‐enrolled students.
  ○ As of 2024, 25 states have expanded their Medicaid programs to allow reimbursement for school‐based services.
- What this study adds
  ○ Medicaid reimbursement expansion for school‐based healthcare increased the supply of federally qualified health clinics providing services within school settings.
  ○ Medicaid reimbursement expansion for school‐based healthcare generally had no significant effect on federally qualified health clinics in other, non‐school settings.

## 1 Introduction

Children from low‐income families face many challenges that impact their health. They are at higher risk for chronic health issues, behavioral problems [1, 2], and developmental delays [3]. Experiencing frequent adverse events due to poverty and community disadvantage makes children from low‐income households more susceptible to various health risks, including mental health issues, substance use, [4] and child maltreatment [5]. Additionally, they often have lower education attainment [6] and diminished long‐term achievement [7]. Further, barriers to accessing healthcare [8], the inability to maintain constant point‐of‐care for healthcare coordination [9], and continuity of care [10] leave children in persistent disadvantaged health situations.

School‐Based Health Centers (SBHCs) provide unique opportunities to address the health needs among children in low‐income families. SBHCs offer the advantage of being conveniently located within school settings, making it easy to reach students and provide consistent healthcare services without any out‐of‐pocket charges. Furthermore, SBHCs primarily serve students from low‐income families. For instance, a national report on SBHCs during 2016–2017 found that about 89% of SBHCs provided services to Title I schools and 70% of SBHC‐served students are eligible for free or reduced‐priced lunch [11]. In addition to serving school‐aged children, SBHCs sometimes extend their healthcare services to the broader community. In 2016–2017, 62% of SBHCs in the U.S. provided health services to populations beyond students in designated schools, including students' family members, students in different schools, non‐attending children, and other community members [11].

The institutions that provide services in SBHCs include hospitals or medical centers, local health departments, private healthcare organizations, school systems, and Federally Qualified Health Centers (FQHCs) [11, 12]. Services offered by SBHCs may vary depending on the site as they cater to the diverse needs of the local community. However, they typically include primary care, mental and behavioral healthcare, nutrition counseling, as well as dental and vision services [13, 14]. As an example, according to the 2022 National Census of School‐Based Health Centers conducted by the School‐Based Health Alliance (SBHA), 73% of SBHCs offered primary care services, and 83% provided behavioral healthcare [15].

Access to healthcare through SBHCs has been associated with improvements in students' health status, including immunization rates [16], reduced overweight and obesity [17], decreased substance use [18], and improved health‐related quality of life [19]. Moreover, studies have found SBHCs’ positive impact on academic performance [20, 21].

Due to the historically free‐of‐charge nature of healthcare services delivered by SBHCs, SBHCs can face financial challenges for their sustainability and the maintenance of quality services. To maintain operations, SBHCs seek funding through various channels, including state and federal government, Medicaid, private insurance, private foundations, partner organizations, and school or school districts [22]. The most common source of funding for SBHCs is federal support through federal grants or sponsorship by FQHCs [14]. FQHCs are community centers designated as Section 330 grantees of the Public Health Service Act and provide healthcare in non‐traditional sites such as schools, homeless shelters, and correctional facilities to maximize access and meet community needs [23, 24]. Per federal funding regulation, FQHCs are mandated to provide comprehensive health services on a sliding fee scale and be located in a designated medically underserved area [25].

The sponsorship of SBHCs through FQHCs has increased over time [26]. FQHCs contribute to the financial sustainability of SBHCs, as they receive funding from the Health Resources and Services Administration [27]. Moreover, FQHCs are well‐suited partners for school districts in managing SBHCs, given their expertise in administering medical offices and obtaining medical supplies [28]. Additionally, FQHCs benefit from higher Medicaid reimbursement rates that reflect the actual costs of providing services than non‐FQHCs [29, 30]. This advantage in reimbursement rates is particularly important considering that SBHCs frequently serve low‐income populations covered by Medicaid.

Medicaid reimbursement has remained a significant financial source for SBHCs or FQHC‐managed SBHCs. However, before 2014, Medicaid could not be billed for all students enrolled in the program receiving healthcare within school settings. The Free Care Rule, enacted in 1997, prohibited Medicaid reimbursement for services provided in schools if the same services were provided free of charge to the general student population (the exception being children with an Individual Education Plan (IEP) or a documented disability). In 2014, the Centers for Medicare & Medicaid Services (CMS) reversed this policy, instead allowing state Medicaid programs to reimburse for health services delivered in schools to all Medicaid‐enrolled students [31].

Though this policy, commonly referred to as the “Free Care Rule reversal,” allowed the option for greater financial support, some states had codified the original policy into their Medicaid state plans. To benefit from the federal policy change, states had to actively amend their state plans or update administration guidance. As of the time of this writing, 25 states have done so and therefore implemented the Free Care Rule reversal [32].

Since schools are required to provide services listed in a student's IEP regardless of Medicaid funding availability, many states and school districts use federal Medicaid funding to cover the expenses of providing these services, releasing some pressure from state and school district's education budgets. According to the Healthy Schools Campaign, eligibility for expanded reimbursement on health services provided to all Medicaid‐enrolled students empowered states to allocate more health resources, offering comprehensive services and covering more vulnerable students [32]. In addition, the Free Care Rule reversal also had positive financial impacts on state Medicaid programs, with states leveraging the reversal experiencing a significant increase in federal Medicaid revenue [33]. The increased federal reimbursement enabled schools to redistribute resources and funds, or bolster school workforce capacity in health services, which can lead to long‐term educational and health benefits [34, 35]. While the reversal of the Free Care Rule presents an opportunity for states and schools to enhance their financial standing and offer more comprehensive medical services to students, states may delay their requests for SBHC expansion for various reasons. Administratively, states that have codified the previous policies into state laws must submit a SPA to CMS. The SPA should include an updated list of eligible medical services and approved Medicaid service providers. After the SPA is approved, states must update their state‐level guidance for school districts, which will detail the revised Medicaid claiming and payment process [36]. Such administrative complexities may cause delays in states' efforts to implement the Free Care Rule reversal.

Especially at a time when children's mental health care needs, in particular, are rising [37], the Free Care Rule reversal could alleviate the financial burden on school health centers and school districts. Therefore, by changing the incentives for schools to house a school‐based health center, expanded eligibility for Medicaid reimbursement may affect the supply side of health services delivered in school‐settings. In this study, we take advantage of state‐level variation in the “take up” of the Free Care Rule reversal to examine the effect of expanding Medicaid to cover school‐based healthcare on the number of healthcare facilities providing services within school settings.

## 2 Methods

### 2.1 Data

The study uses two primary data sources. The first data source is the number of FQHCs delivering healthcare services within school settings. These data come from the Uniform Data System on the Health Resources and Services Administration (HRSA) [38]. The HRSA provides a national longitudinal dataset on federally qualified health center programs, categorizing them by various locations, including schools. This feature enables us to identify the school‐based FQHCs across states during the study period. These data span 2012–2020; we end the analysis period in 2020 due to the nature of the relationship between children and schools changing as a result of the COVID‐19 pandemic. Specifically, decisions about whether to add a school‐based FQHC were likely halted in 2020, whereas FQHCs that existed in 2020 were likely decided before 2020.

We combine these data on school‐based health clinics with state policy variables documenting whether states implemented Medicaid SBHC expansion to cover healthcare services delivered in schools. To implement SBHC expansion, several states were required to submit a State Plan Amendment (SPA) to CMS, while others could initiate the expansion process immediately without requiring a SPA. We use the Healthy Schools Campaign tracker [39]—which documents state efforts on school SBHC expansions—and confirm with our own examination of state Medicaid plan documents and legislative records.

There are three types of SBHC expansions to reflect take‐up of the Free Care Rule reversal: First, most states that have expanded have fully expanded Medicaid reimbursement to cover services to eligible students, offering comprehensive policies that include all medically necessary services outside of an IEP for all Medicaid‐enrolled students. Second, some states offer reimbursement for services outside of an IEP for specific student groups. For example, some states expanded their coverage to include all medical services for Medicaid‐enrolled students with a 504 plan, Individualized Health Plan, or Behavior Intervention Plan [32]. Finally, some states cover reimbursement for partial services, including additional behavioral and physical health services outside of an IEP, for all Medicaid‐enrolled students. For example, Georgia expanded its school Medicaid program in 2021 to cover nursing services for all Medicaid‐enrolled students, and Arkansas and Missouri both cover behavioral health services. We consider the first and second types to be a “full‐service expansion” and the third type to be “partial service expansions,” since we are interested in whether offering reimbursement for a set of comprehensive services to Medicaid‐enrolled children influences the supply of FQHCs. Table 1 documents our coding of state SBHC expansions to school services, and the year in which the state expanded its SBHC program. States that did not initiate the expansion process or expanded after 2020 when the study period ends were designated as the control group.

**TABLE 1:** Dates of state Medicaid SBHC expansion.

Full expansion	Year of full expansion	Partial expansion	Year of partial expansion
California*	2020	Arkansas	2015
Colorado*	2020	Missouri	2018
Connecticut*	2017		
Florida*	2020		
Kentucky*	2019		
Louisiana*	2015		
Massachusetts*	2019		
Michigan*	2019		
Nevada*	2019		
New Hampshire	2017		
North Carolina*	2019		
South Carolina	2016		
Washington	2015		

*Note*: Several states expanded their Medicaid programs to accept school services after our study period (ending in 2020). These are considered control states in our analysis and include: Arizona* (2021), Georgia* (2021), Illinois* (2023), Indiana* (2023), Minnesota (2021), New Mexico* (2023), North Dakota (2021), Oregon* (2023), Tennessee (2023), and Virginia* (2023). The state of Washington is a special case. Local schools can contract with Managed Care Organizations (MCOs) to receive reimbursement for non‐Individual Education Plan (IEP) services. Although it is not an expansion through the free care policy reversal, it does create an opportunity to increase reimbursement by MCOs, and as of May 2023, 7 out of 9 districts are contracted. *Indicates states that expanded via a state plan amendment.

Abbreviation: SBHCs, school‐based health centers.

### 2.2 Statistical Analysis

We compare the number of federally qualified school‐based health clinics in states that expanded their Medicaid programs to cover school‐based services to the number in states that did not expand, both before and after state Medicaid SBHC expansion. Because states expanded their Medicaid programs to cover school‐based services at different times, we employ a staggered difference‐in‐differences taking the following form:

(1) $$\;Y_{\mathrm{sy}}=\beta_{0}+\beta_{1}{\text{Expansion}}_{\mathrm{sy}}+\gamma_{y}+\delta_{s}+\lambda^{\prime }X_{\mathrm{sy}}+\varepsilon_{\mathrm{sy}}$$

where *Y* is the number of school‐based FQHCs in state *s* during year *y*. Expansion_*sy*_ is a binary variable equal to 1 if state *s* expanded its SBHC program to cover school‐based healthcare services during year *y*, and zero otherwise. The parameter of interest is $\beta_{1}$, which represents the effect of the SBHC expansion. Models include state ($\delta_{s}$) and year ($\gamma_{y}$) fixed effects and the time‐varying state‐level covariates ($\lambda^{\prime }X_{\mathrm{sy}}$) described below. Due to the challenges of estimating dynamic effects and using previously treated states as a comparison group in OLS; however, we also employ the Callaway and Sant'Anna (2021) estimator [40].

We adjusted the model for variables that may be correlated with both the number of school‐based health centers in a state and whether the state decided to expand its Medicaid coverage to schools after CMS reversed the Free Care Rule. These variables include the state‐level unemployment rate, the percentage of students eligible for the National School Lunch Program, Medicaid expansion to low‐income adults under the Affordable Care Act (ACA), and the amount of funding received by school‐based FQHCs received as part of the ACA in the form of Capital Development Grants, including SBHC capital grants. State‐level annual unemployment rates and number of students eligible for the School Free Lunch Program were sourced from the University of Kentucky Center for Poverty Research [41], while the state‐level school‐aged child population was obtained from CDC WONDER [42]. The state‐level rate of participation in the School Free Lunch Program was calculated by dividing the state's annual School Free Lunch Program population by the state's annual school‐aged child population. Finally, the ACA Medicaid expansion status was obtained from Bullinger et al. [43], and the ACA funding for SBHCs came from HRSA.

The primary assumption of a difference‐in‐differences approach is that the comparison group—states that did not expand—is a good counterfactual for the treatment group—states that did expand. In other words, absent the SBHC expansions, the number of school‐based clinics would have continued the same trends in both expansion and non‐expansion states. To provide evidence supporting this “common trends” assumption, and to examine potential dynamic effects following the expansion, we also estimate event study analyses, which offer a more flexible version of Equation (1). In total, our analysis consists of 50 states plus Washington D.C., measured for 9 years. All statistical analyses were conducted in Stata version 18, and *p* < 0.10 was used to determine statistical significance.

### 2.3 Robustness Checks

We conducted several sensitivity analyses to ensure the robustness of our results. First, we include the number of school‐based FQHC “look‐alikes” with that of school‐based FQHCs. FQHC look‐alikes refer to health centers that meet the requirements of the HRSA Health Center Program but do not receive its funding [44]. While not officially designated as FQHCs, these look‐alikes share similarities with FQHCs, such as the population they serve, the enhanced reimbursement structure, and the services they provide [45]. Therefore, we expect that school‐based FQHC look‐alikes would experience a similar influence to school‐based FQHCs following the expansion of school Medicaid services. Importantly, we only have data on look‐alike sites from 2016 through 2020, so the sample size is smaller when using these data.

Second, as a falsification test, we replace the number of school‐based FQHCs with the number of FQHCs in alternative location settings, such as correctional facilities, domestic violence shelters, hospitals, nursing homes, and “other” settings. Since the SBHC expansion would not necessarily impact the number of FQHCs outside of schools, we expect to find no effect of the expansions on the number of FQHCs delivering services in alternative settings. This test also provides evidence on whether broader FQHC policies, such as ACA funding for FQHCs, are likely driving the results.

Finally, we test the robustness of the results to various functional forms and study periods. Specifically, we log transform the outcome, measure the FQHCs as a rate per 100,000 school‐aged children, and drop observations in 2020 from the analysis altogether.

### 2.4 Limitations

Our analysis has some limitations. First, previous literature on SBHCs has used the National Census of School‐Based Health Centers from the SBHA to measure the number of SBHCs [26, 46, 47]. However, this dataset does not distinguish between clinics not responding to the Alliance's survey and clinics that have closed. In other words, survey non‐response and missing data are measured the same. While the response rate to the Alliance's survey is high in recent years [47], identifying existing SBHCs in earlier years is more challenging. Since we are measuring the *existence* of school‐based healthcare delivery over time, it is important to have consistently measured variables throughout the study period. Specifically, we estimate that FQHC delivering services within schools consist of approximately 60% of all school‐based health centers documented in 2016–2017 by the SBHA. Therefore, our estimates likely do not capture the full census of school‐based health centers, per the SBHA definition. Because of federal reporting requirements among FQHCs, FQHC data are more likely to be appropriately measured across states over time, but they may miss less formal arrangements for school‐based care or school‐based health centers that do not serve underserved areas. Furthermore, we would be counting existing clinics that shift their administration to FQHCs as new clinics, when in reality, they just have a new qualification. Nonetheless, this change may be due to the new financial incentives that were brought about by the free care rule reversal.

Due in part to the different relationship between children and their schools and healthcare services during the COVID‐19 pandemic, we end our study period in 2020. Many states are continuing to expand their Medicaid programs to authorize reimbursement for school‐based services. Therefore, we view our results as early evidence of the effects of these policies.

Finally, our data are measured at the state‐year level. Although this level of aggregation is blunt, the policy change is at the state level, meaning any analysis using lower levels of aggregation would need to cluster standard errors at the state level, implying the same effective sample size for statistical inference.

## 3 Results

### 3.1 Main Results

During the study period spanning 2012–2020, 13 states implemented full coverage for all health services to Medicaid‐eligible students, while three states implemented partial coverage for specific health services (see Table 1). In our analysis, states that expanded coverage after the study period ends in 2020 were classified as never‐treated states. These states include Arizona (2021), Georgia (2021), Illinois (2023), Indiana (2023), Minnesota (2021), New Mexico (2023), North Dakota (2021), Oregon (2023), Tennessee (2023), and Virginia (2023).

We first consider the effect of a full‐service expansion. In this analysis, states that fully expanded Medicaid reimbursement for Medicaid‐eligible students are in the treatment group, while partial and untreated states serve as the comparison group. In a second analysis, we consider the treatment group to be states with either full or partial coverage, with untreated states as the comparison group.

**FIGURE 1:**
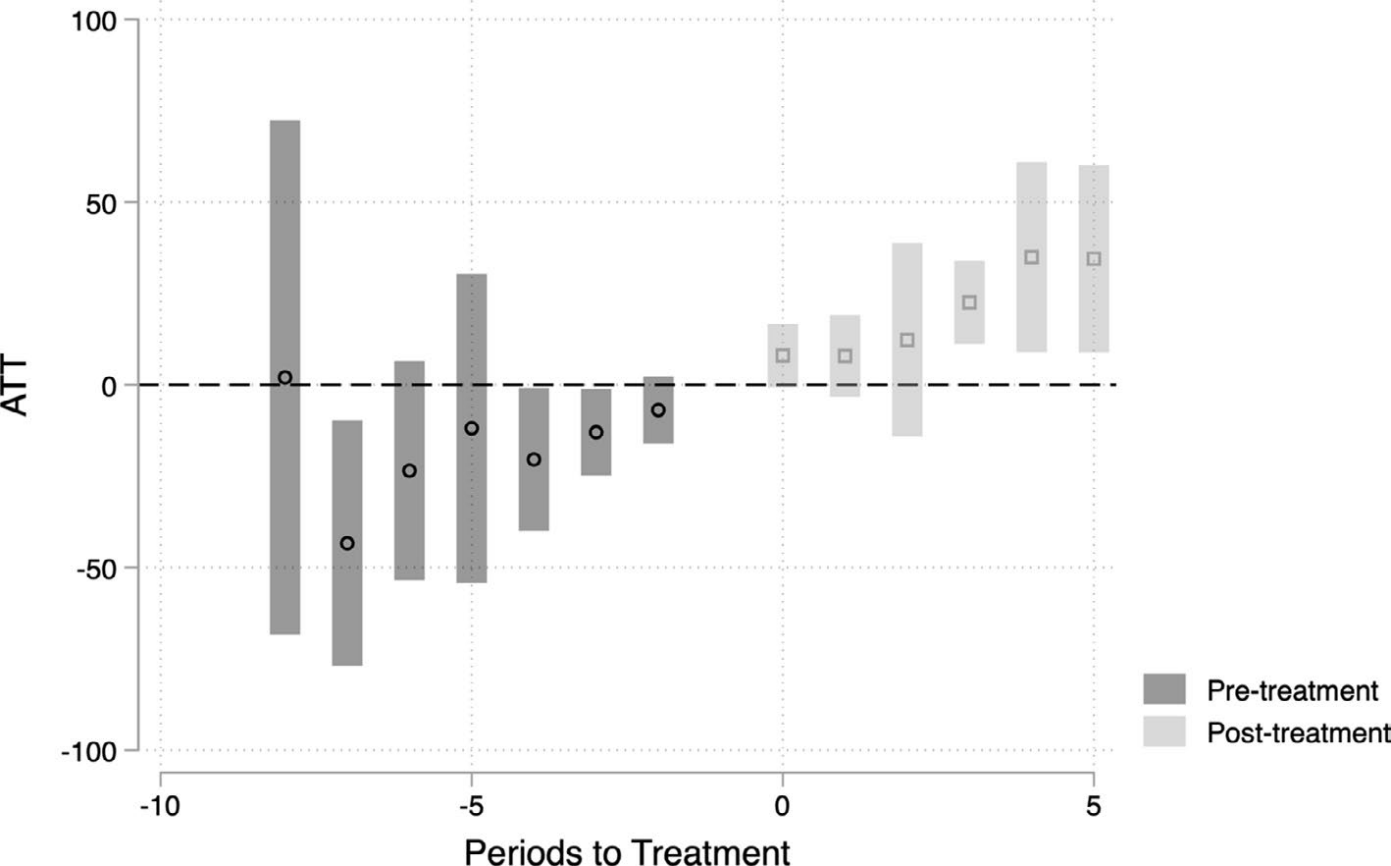
Effect of full school Medicaid expansion on the number of school FQHCs. FQHC, federally qualified health center; ATT, average treatment effect for the treated group. Data come from Health Resources and Services Administration (HRSA) 2012–2020 and Healthy Schools Campaign. Results reflect column (2), panel B of Table 2. Bars reflect the 95% confidence interval.

Figure 1 demonstrates the effect of full‐service expansion on the number of school‐based FQHCs while partial and no expansion states are the comparison group. As indicated by the point estimates and accompanying 95% confidence intervals, states that fully expanded comprehensive services have slightly lower numbers of SBHCs than states in the comparison group in the years leading up to each state's expansion date. Upon SBHC expansion for Medicaid‐eligible students, the number of school‐based FQHCs increased, and the increase grew larger over time.

Table 2 summarizes the results. Specifically, in Panel A we show the results when using OLS, and in Panel B we show the Callaway & Sant'Anna (2021) estimates. Column 1 of Panel A suggests that the full‐service expansion increased school‐based FQHCs by 15.25 centers (*p* < 0.10). When controlling for state‐level unemployment rate, participation in the National School Lunch Program, ACA Medicaid expansion status, ACA SBHC funding, and the school‐aged child population, fully expanding to cover all services for Medicaid‐eligible students (Column 2) is associated with an increase in the number of school‐based FQHCs by 15.83 (*p* < 0.10). When compared to the baseline mean of 34.8, this estimate represents a relative increase of about 45%. When including the partial expansion states in the treatment group (Columns 3 and 4), the results are larger in magnitude but less precise. The point estimates indicate that about 16–17 more school‐based FQHCs exist after the Medicaid expansion to cover school‐based services. Appendix S1 reports the full set estimates for the OLS estimates.

**TABLE 2:** Effects of Medicaid school expansions on the number of FQHCs delivering healthcare services within school settings.

	Full expansion		Full or partial expansion	
	(1)	(2)	(3)	(4)
Panel A: OLS				
SBHC expansion	15.25*	15.83*	15.61	16.82*
	(0.064)	(0.051)	(0.106)	(0.080)
Covariates	*N*	*Y*	*N*	*Y*
Mean of *Y* before free care rule reversal	34.8	34.8	31.9	31.9
*N*	459	459	459	459
Panel B: callaway and sant'anna (2021)				
SBHC expansion	9.02	13.42**	8.98	7.05
	(0.169)	(0.035)	(0.111)	(0.365)
Covariates	*N*	*Y*	*N*	*Y*
Mean of *Y* before free care rule reversal	34.8	34.8	31.9	31.9
*N*	459	451	459	454

*Note*: Data are from Health Resources and Services Administration (HRSA), 2012–2020. Estimates in panel A are obtained using ordinary least squares (OLS). Estimates in panel B are obtained using staggered treatment difference‐in‐differences by Callaway & Sant'Anna (2021). Covariates include: Affordable Care Act (ACA) Medicaid expansion to low‐income adults, unemployment rate, the percent of children eligible for the National School Lunch Program, the amount of funding for school‐based health centers received as part of the Affordable Care Act, the school‐aged population and the quadratic of the school‐aged population. *p* values in parentheses.

Abbreviations: FQHC, federally qualified health center; SBHC, school‐based health centers.

* *p* < 0.10.

** *p* < 0.05.

*** *p* < 0.01.

Panel B reports the Callaway & Sant'Anna (2021) estimates, which better account for effects growing or shrinking over time and for the fact that previously treated states should not serve as comparison states, as is the case with OLS. These results are more conservative; as shown in Column 1, without covariates, SBHC expansion is suggested to increase school‐based FQHCs by about 9, but this result is not statistically significant. When including pre‐treatment covariates, Column 2 shows that full SBHC expansion increased the number of school‐based FQHCs by 13.42 (*p* < 0.05). Relative to the baseline mean of 34.8, this increase reflects a rise of 39%. As in the OLS results, when including partial expansion states in the treatment group, Columns 3 and 4 report attenuated estimates of about 8 or 9 per state year, but these estimates are not statistically different from zero. In sum, these results indicate an increase in the supply of FQHCs delivering healthcare services in school settings following the expanded Medicaid coverage to school‐based care.

### 3.2 Sensitivity Analyses

We first conduct a falsification test estimating the effect of the expansion on non‐school based clinics, relying on the Callaway & Sant'Anna (2021) estimates. Specifically, we compared the number of FQHCs in alternative settings in SBHC expansion relative to non‐expansion states using Equation (1). Table 3 reports the results on the number of FQHCs in correctional facilities, domestic violence shelters, hospitals, and nursing homes, and “other” settings. We observe no statistically significant effect on FQHCs in these settings as a result of Medicaid coverage to school‐based healthcare, with the exception of a marginally significant increase in FQHCs in correctional facilities of much smaller magnitude (both absolute and relative) than those observed for school‐based FQHCs. These results strengthen the main findings that the financial incentive for school‐based coverage had a particular effect on access to school‐based clinics.

**TABLE 3:** Effect of full medicaid school expansions on FQHCs delivering healthcare services within other settings.

	(1)	(2)	(3)	(4)	(5)
	Correctional facilities	Domestic violence shelters	Hospitals	Nursing homes	“Other”
SBHC expansion	0.16*	−0.39	−0.22	0.03	4.37
	(0.077)	(0.300)	(0.338)	(0.934)	(0.526)
Mean of *Y* before free care rule reversal	0.8	1.9	2.5	1.5	186.6

*Note*: Data are from Health Resources and Services Administration (HRSA), 2012–2020 (*N* = 442). Estimates obtained using staggered treatment difference‐in‐differences by Callaway & Sant'Anna (2021). Covariates include: Affordable Care Act (ACA) Medicaid expansion to low‐income adults, unemployment rate, the percent of children eligible for the National School Lunch Program, the amount of funding for school‐based health centers received as part of the Affordable Care Act, the school‐aged population and the quadratic of the school‐aged population. *p* values in parentheses.

Abbreviations: FQHC, federally qualified health center; SBHC, school‐based healthcare center.

* *p* < 0.10.

** *p* < 0.05.

*** *p* < 0.01.

Appendix S1 reports additional sensitivity analyses. Here, we included “look‐alike” FQHCs in the primary outcome measure. These health centers meet the requirements of the HRSA Health Center Program but do not receive its funding. As shown in Panel A of Appendix S1, when we include these look‐alike sites, the results are similar.

When we log‐transform the outcome (making all 0 observations 0.001), the results show no significant relationship between school‐based Medicaid expansion and school FQHCs, though the coefficients are negative. These results are reported in Panel B of Appendix S1. Panel C shows the effects when using number of school‐based FQHCs per 100,000 school‐aged children, which are also imprecise nulls. Finally, Panel D of Appendix S1 shows that when we omit year 2020, the results are substantively similar though slightly attenuated. In sum, although the precision and magnitude of the results are sensitive to specification, functional form, and sample inclusion, the sensitivity checks generally support the main finding that Medicaid expansions to school‐based healthcare increased the financial incentives for schools to have health clinics in them, and these incentives have been effective.

## 4 Discussion

Children in the United States face many unmet healthcare needs. In particular, mental healthcare treatment among school‐aged children is very low relative to need, and this need only exacerbated during the COVID‐19 pandemic [37]. One potential solution for meeting these unmet needs is to increase access to mental, behavioral, and primary/preventive care via educational systems. The resources of schools and educational systems, however, are already stretched thin. By reversing the Free Care Rule in 2014, CMS allowed state Medicaid programs to begin covering healthcare provided to Medicaid‐enrolled children, opening the door for a more sustainable funding structure to providing health services and mental health treatment to students.

In this paper, we find that expanding Medicaid coverage to comprehensive healthcare services provided within schools increased the supply of clinics offering healthcare services in school settings by approximately 40%. In other words, the financial incentives for school health services increased student access to care in the form of more federally qualified school‐based clinics. In 2019, the average school‐based FQHCs served about 10,213 Medicaid‐covered children. With an increase of 13.42 school‐based FQHCs, our results imply an increase of approximately 137,000 more Medicaid children served by school‐based FQHCs in each expansion state as a result of these SBHC expansions.

The results in Figure 1 demonstrate that the increase in clinics is growing over time. Given that our results included only a few post‐SBHC expansion years for several states, it is possible that the effects will be even larger in a few more years. This growing effect size over time may be due, in part, to the administrative procedures that state departments must undertake to initiate the Medicaid reimbursement process for school districts, or the substantial support and resources needed to navigate the new billing structure and adapt to this policy change. Schools may need to hire more approved Medicaid service providers to reimburse for expanded services offered to students. These changes take time to implement, allowing schools to gradually enhance their billing processes and staffing.

Since the data we employ are strictly FQHCs offering services in school settings, they may not include less formal partnerships between schools and community providers or health services provided via school‐employed providers (e.g., school nurses). Since these less formal mechanisms for bringing health services to students are administratively less burdensome and therefore also likely increasing, our estimates are likely a lower bound of the effects of this coverage on access to school‐based health services. Alternatively, if existing healthcare centers became FQHCs as a result of this Medicaid reimbursement, we would be overestimating the effects.

These findings are important for ongoing policy discussions and debates. For example, in 2022, the federal legislation, the Bipartisan Safer Communities Act, implemented several provisions regarding Medicaid and school‐based services. For example, Title I of the law is focused on providing guidance to state Medicaid agencies and local educational entities to provide services to Medicaid‐covered students in school‐based settings. The legislation also substantially increased funding to expand school‐based mental health services and launched a technical assistance center bringing together CMS and the Department of Education. Our findings imply these provisions are likely to have a stronger influence in states that already opted into the Free Care Rule reversal. Furthermore, our findings highlight that states that have not yet expanded their coverage to include school‐based health services are missing out on an opportunity to draw down additional federal funding for school health services and expand the types of health services and providers that are eligible for Medicaid reimbursement.

Finally, the COVID‐19 pandemic illuminated the critical role that schools play in the health of children. Especially for children in underserved communities, schools may be the only source of healthcare. CMS has offered a new source of funding for schools to deploy health services, and it has been effective at increasing the supply of school‐based health clinics. Future research should examine whether this expanded coverage of school‐based health services has affected child health and reduced health disparities.

## Conflicts of Interest

The authors declare no conflicts of interest.

## Supporting information


**Appendix S1.** Supporting Information.
