# Supplementary material for: The Effects of Medicaid Coverage for School‐Based Health Services on the Supply of School‐Based Healthcare Clinics: Evidence From the “Free Care Rule” Reversal and FQHCs
Source: Health Serv Res. 2025 Feb 12;60(Suppl 2):e14452. doi: 10.1111/1475-6773.14452 (PMC12047691; doi:10.1111/1475-6773.14452)
Supplement: Supplementary file 1 — Appendix S1. Supporting Information. [file HESR-60-0-s001.docx]

**Appendix A. Full OLS Results on Number of FQHCs Delivering Healthcare Services within School Settings**

|  | Full Expansion | |  | Full or Partial Expansion | | |  |
| --- | --- | --- | --- | --- | --- | --- | --- |
|  | (1) | (2) |  | (3) | (4) |  |  |
| SBHC Expansion | 15.25* | 15.83* |  | 15.61 | 16.82* |  |  |
|  | (0.064) | (0.051) |  | (0.106) | (0.080) |  |  |
| ACA Expansion |  | 1.13 |  |  | 0.98 |  |  |
|  |  | (0.750) |  |  | (0.780) |  |  |
| Unemployment rate |  | -1.87 |  |  | -2.08 |  |  |
|  |  | (0.162) |  |  | (0.135) |  |  |
| NSLPFreeRate |  | -0.03 |  |  | -0.07 |  |  |
|  |  | (0.962) |  |  | (0.920) |  |  |
| ACA SBHC Funding (in thousands of dollars) |  | -0.27 |  |  | -0.28 |  |  |
|  |  | (0.358) |  |  | (0.356) |  |  |
| School-aged Child Population |  | -0.00 |  |  | -0.00 |  |  |
|  |  | (0.137) |  |  | (0.130) |  |  |
| School-aged Child Population^2^ |  | 0.00* |  |  | 0.00* |  |  |
|  |  | (0.096) |  |  | (0.090) |  |  |
| Covariates | N | Y |  | N | Y |  |  |
| Mean of Y Before Free Care Rule Reversal | 34.8 | 34.8 |  | 31.9 | 31.9 |  |  |
| N | 459 | 459 |  | 459 | 459 |  |  |
| Abbreviation: OLS, Ordinary Least Squares. SBHC, School-Based Healthcare Center. NSLP, National School Lunch Program. ACA, Affordable Care Act.  Notes: Data are from Health Resources and Services Administration (HRSA), 2012-2020. Estimates obtained using two-way fixed effects. Regressions include state and year fixed effects and the following covariates: Affordable Care Act (ACA) Medicaid expansion to low-income adults, unemployment rate, the percent of children eligible for the National School Lunch Program, the amount of funding for school-based health centers received as part of the Affordable Care Act, and the quadratic of the school-aged population. Robust standard errors are clustered at the state-level. p-values in parentheses.  * p<0.10, ** p<0.05, *** p<0.01 | | | | | | | |

| **Appendix B. Sensitivity Analyses** |  |  |  |  |  |
| --- | --- | --- | --- | --- | --- |
|  | Full Expansion | |  | Full or Partial Expansion | |
|  | (1) | (2) |  | (3) | (4) |
| **Panel A: Including Look Alike Sites (2016-2020)** | | | | | |
| SBHC-Expansion | 15.01* | 16.32* |  | 13.21 | 14.76 |
|  | (0.066) | (0.051) |  | (0.147) | (0.120) |
| Covariates | N | Y |  | N | Y |
| Mean of Y Before Free Care Rule Reversal | 50.1 | 50.1 |  | 46.1 | 46.1 |
| N | 255 | 255 |  | 255 | 255 |
|  |  |  |  |  |  |
| **Panel B: Log(Number of FQHCs Delivering Healthcare Services within School Settings)** | | | | |  |
| SBHC-Expansion | -0.24 | -0.21 |  | -0.36 | -0.33 |
|  | (0.470) | (0.530) |  | (0.317) | (0.397) |
| Covariates | N | Y |  | N | Y |
| N | 459 | 459 |  | 459 | 459 |
|  |  |  |  |  |  |
| **Panel C: Number of FQHCs Per 100,000 School-Aged Children** | | | | |  |
| SBHC-Expansion | 0.74 | 0.90 |  | 0.78 | 0.98 |
|  | (0.578) | (0.493) |  | (0.613) | (0.523) |
| Covariates | N | Y |  | N | Y |
| Mean of Y Before Free Care Rule Reversal | 3.1 | 3.1 |  | 2.97 | 2.97 |
| N | 459 | 459 |  | 459 | 459 |
| **Panel D: Number of FQHCs Delivering Healthcare Services within School Settings, No 2020** | | | | | |
| SBHC-Expansion | 12.48 | 11.81 |  | 13.21 | 12.31 |
|  | (0.116) | (0.116) |  | (0.170) | (0.182) |
| Covariates | N | Y |  | N | Y |
| Mean of Y Before Free Care Rule Reversal | 34.8 | 34.8 |  | 31.9 | 31.9 |
| N | 408 | 408 |  | 408 | 408 |
| Abbreviation: FQHC, Federally Qualified Health Center. SBHC, School-Based Healthcare Center.  Notes: Data are from Health Resources and Services Administration (HRSA), 2012-2020. Estimates obtained using staggered treatment difference-in-differences, estimated using OLS. Covariates include: Affordable Care Act (ACA) Medicaid expansion to low-income adults, unemployment rate, the percent of children eligible for the National School Lunch Program, the amount of funding for school-based health centers received as part of the Affordable Care Act, the school-aged population and the quadratic of the school-aged population. p-values in parentheses.  * p<0.10, ** p<0.05, *** p<0.01 | | | | | |
